# Telemedicine in the Clinical Care of Neglected Tropical Diseases: A Scoping Review

**DOI:** 10.1371/journal.pntd.0012431

**Published:** 2025-04-08

**Authors:** Fernanda Gonçalves Ferreira Salvador, Mayumi Duarte Wakimoto, Claudia Cristina Jardim Duarte, Luis V Lapão, Henrique Silveira, Cláudia Maria Valete

**Affiliations:** 1 Global Health and Tropical Medicine, GHTM, Associate Laboratory in Translation and Innovation Towards Global Health, LA-REAL, Institute of Hygiene and Tropical Medicine, Universidade Nova de Lisboa, Lisbon, Portugal; 2 Laboratory of Clinical Research and Surveillance in Leishmaniasis, Evandro Chagas National Institute of Infectious Diseases, Oswaldo Cruz Foundation, Rio de Janeiro, Brazil; 3 Health Surveillance Service, Evandro Chagas National Institute of Infectious Diseases, Oswaldo Cruz Foundation, Rio de Janeiro, Brazil; 4 Department of Otorhinolaryngology and Ophthalmology, School of Medicine, Federal University of Rio de Janeiro, Rio de Janeiro, Brazil; Mizan-Tepi University, ETHIOPIA

## Abstract

**Background:**

The Neglected Tropical Diseases (NTDs) represent a global public health problem. Telemedicine uses telecommunications to deliver remote healthcare services when patients and providers are separated by distance. Using digital health technologies to offer medical care remotely to NTDs can be an important strategy for reducing inequalities in access but is challenging in low-and middle-income settings. The objective of the current review was to identify and summarize international evidence on the use of telemedicine for clinical care of patients with NTDs around the world based on a scoping review protocol.

**Methodology/Principal Findings:**

A total of 422 articles were extracted from the databases MEDLINE/PubMed, Web of Science and Scopus, and after removing 129 duplicates, 285 studies were excluded because they did not meet the eligibility criteria. A total of 8 articles were included for data extraction, half of them published after 2021 (n=4). Four studies were focused on dermatological diseases, like leprosy and leishmaniasis, and the other diseases found were dengue (n=2), trachoma (n=1) and cysticercosis (n=1). Most telemedicine interventions in NTDs involved Primary Health Care teams (n=5). Studies that evaluated the accuracy of remote diagnosis demonstrated good effectiveness for leprosy, trachoma and complications of neurocysticercosis. There was a reduction in the need for specialized in-person medical consultations with telemedicine for the care of dengue fever and some dermatological NTDs; and an improvement in the quality of clinical monitoring of cutaneous leishmaniasis using mobile health was demonstrated.

**Conclusions/Significance:**

Although we observed a small recent increase in academic research on the theme, there is a gap in recommendations for the clinical management of NTDs through telemedicine in the literature. The feasibility and potential for telemedicine-based NTDs diagnosis and treatment have been demonstrated in only a few settings/countries, revealing that this resource is still largely underutilized.

## Author Summary

Neglected Tropical Diseases (NTDs) represent a global public health problem with great socioeconomic impact. Using digital health technologies to provide medical care remotely can be an important strategy for reducing inequalities. The objective of this scoping review was to identify and summarize international evidence on the use of telemedicine in the clinical care of patients with NTDs. A total of 8 articles were included for data collection as they met all our search criteria, half of them published after 2021 (n=4). Most telemedicine interventions in NTDs involved Primary Health Care teams (n=5). Studies that evaluated the accuracy of remote diagnosis demonstrated good effectiveness for leprosy, trachoma and complications of neurocysticercosis. There was a reduction in the need for specialized face-to-face medical consultations with telemedicine to treat dengue fever and some dermatological NTDs, and an improvement in the quality of clinical monitoring of cutaneous leishmaniasis through mobile health was reported. Although we have seen a recent small increase in academic research on the topic, there is a gap in recommendations for the clinical management of NTDs through telemedicine in literature, revealing that this resource is still largely underutilized.

## Introduction

Neglected Tropical Diseases (NTDs) are estimated to affect more than a billion people in poor and vulnerable regions of the world, but they occupy a disproportionate space in the priorities of the international agenda, reflected in low investments in research, therapeutic strategies and elimination/control actions [1,2]. In addition to early mortality, disabilities caused by NTDs perpetuate cycles of social exclusion due to unemployment and low education and are strongly related to socioeconomic conditions such as irregular access to drinking water, basic sanitation and adequate housing. The control of these diseases is formally recognized as a goal of global action in the Sustainable Development Goals (SDGs) defined by the United Nations (UN) and the development of mechanisms to expand universal access to health is one of the main strategies recommended by the World Organization World Health Organization (WHO) to combat NTDs [1].

NTDs are prevalent in low- and middle-income countries (LMIC) that often have low coverage by health systems or irregular distribution of doctors between territories. Using digital health technologies to provide medical care remotely is an important strategy for reducing inequalities in access to healthcare [3,4]. But, despite recent advances, this potential for remote assistance still appears to be scarcely explored [5]. Many NTDs are endemic in rural and remote areas and may require rapid guidance for specific clinical management (e.g. snakebite envenoming), with great potential for the use of these strategies. In other situations, the low prevalence and/or high complexity of the pathology may require highly trained focal specialists for diagnosis, and teleconsultations with distant hospital centers can optimize timely care and early treatment of NTDs, reducing the time between the onset of symptoms and specialized clinical care.

The Covid-19 pandemic triggered a rapid expansion in the provision of remote care via telemedicine, making it an indispensable resource for the healthcare sector [6]. The growth of successful experiences in Digital Health can be observed especially in high-income countries, but the legal regulation of this practice is heterogeneous, and its incorporation has been slower in LMIC due to low internet connection and infrastructure barriers [7]. Health systems have different levels of technological capacity to incorporate innovations, and many poor countries will need sustainable financial support and international technical cooperation to implement initiatives in this field.

Telemedicine uses telecommunications and information systems to deliver remote healthcare services when patients and providers are separated by distance [3]. Different strategies can be useful during the therapeutic journey of NTDs care via telemedicine, such as synchronous telecare between professionals and patients in real time, or the exchange of opinions between experts from distant services for difficult cases. Remotely sending data, such as images and videos, can also be a simple and useful way to carry out an asynchronous teleconsultation, and the choice of the most appropriate resources will depend on the clinical situation and local context [8].

Despite the recent wave of interest in technological innovations on health [9], the literature about successful experiences of remote clinical care via telemedicine in contexts of populations affected by NTDs is unclear. To the best of our knowledge no previous study has carried out to systematically map and categorize experiences in individual remote clinical care for NTDs. This review seeks to shed light on the current global landscape of this topic, which will be useful for NTDs clinicians and public health decision-makers.

## Methods

### Research questions and scope

The goal of this scoping review is to map and summarize existing literature on telemedicine applied to NTDs to address the review question: “what is the current state of scientific evidence on the use of telemedicine in the clinical care of patients with NTDs worldwide?”

To answer the study question, we adopted the strategy Population (P), Concept (C) and Context (C), the conceptual mnemonic model PCC [11]. The Population was defined as people affected by NTDs; the Concept as telemedicine interventions for remote clinical care; and the Context as all health settings worldwide. We analyze and organize the findings in ways that highlight potential benefits for the diseases identified. We also suggest ways to conduct future studies.

### Definitions

The following subsections provide definitions and explanations of the key terms used throughout this paper.

For the review we considered the list of 21 NTDs and disease groups currently defined and prioritized by WHO [12], with the aim of delimiting the scope of this research.

There are many conceptual definitions of telemedicine in literature. In this study we assume a WHO definition [8] which encompasses the following activities: 1. consultations between a remote person and a healthcare professional; 2. remote monitoring of the person’s health or diagnostic data by the provider; 3. transmission of medical data (e.g. images, notes and videos) to the healthcare provider; and 4. case management consultations between healthcare providers.

We report that, due to the characteristics of the regions studied, the team considered all available means of communication, from videoconferences to regular calls by telephone, for example, as acceptable telemedicine resources.

### Protocol and registration

This scoping review was conducted in accordance with the Joanna Briggs Institute (JBI) methodology for Scoping Reviews [10] and followed the methodological framework suggested by the Preferred Reporting Items for Systematic Reviews and Meta-Analyses for Scoping Review (PRISMA–ScR) [11]. The research protocol was registered on the Open Science Framework plataform (OSF) in March 2024 and can be accessed in: https://doi.org/10.17605/OSF.IO/XAKF5. The team was composed of academic researchers, clinical health professionals, and digital health specialists.

### Eligibility criteria

The inclusion criteria were: 1. Telemedicine for individual clinical care; 2. Report at least one NTDs from the WHO list; and 3. Published between January 2000 and March 2024.

No language restrictions were applied. Systematic and scoping reviews, case studies, book chapters, editorials, grey literature and media sources were excluded.

### Search strategy

Three databases and interfaces were chosen for their reliability and ease of searching with extensive MeSH terms: MEDLINE/PubMed, Web of Science and Scopus. An advanced search in the three selected databases was performed using Boolean operators to identify articles of interest published before March 26, 2024 (date of last retrieval).

**Fig 1:**
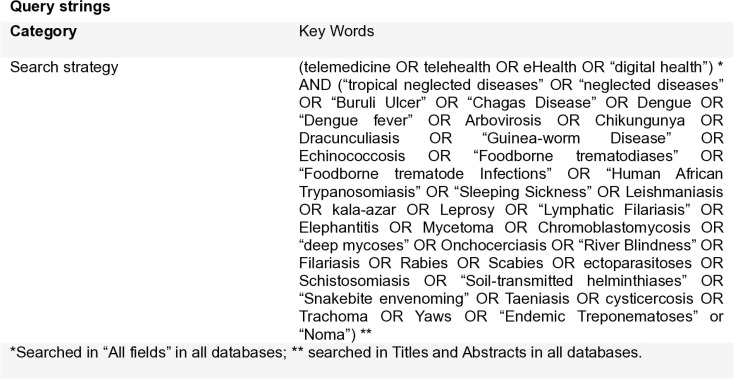
Search strategy.

The keywords and their variations were defined by the working group based on literature review and sensitivity pre-test of the terms in retrieving relevant articles. Combining these groups, we generate keywords lists for two categories (Fig 1).

The expression “telehealth” was also used in our primary search strategies, however only studies that carried out activities within the scope of telemedicine mentioned above were selected [8]. The same criteria were applied to studies retrieved by the terms Digital Health and eHealth (electronic health).

### Selection process

Titles and abstracts were selected by two independent reviewers for evaluation according to the inclusion criteria using the Rayyan automation tool [13]. In the same way, the selected articles were read in full and classified. The steps were blinded to reduce bias. All disagreements that arose between the reviewers at each stage of the study selection process were resolved by a third reviewer.

### Data extraction and analysis

Data extraction was performed independently by the reviewers. An instrument for evidence synthesis was created in Microsoft Excel after a extraction form was pilot tested by the team, to ensure alignment in capture.

Included studies were divided into the following categories: the characteristics of the articles (authors, year, country of the studied population, country income, main NTD/disease, study design, study focus, sample); contextual factors (communication technology used, type and duration of the telemedicine intervention, rural or urban area, telemedicine care providers involved, team responsible for the telemedicine initiative,clinical applicability) and main outcomes/The major findings were analysis using a thematic descriptive approach. The methodological quality of the studies was not thoroughly evaluated and no critical appraisal of individual sources of evidence was carried out.

## Results

### Eligible records

**Fig 2:**
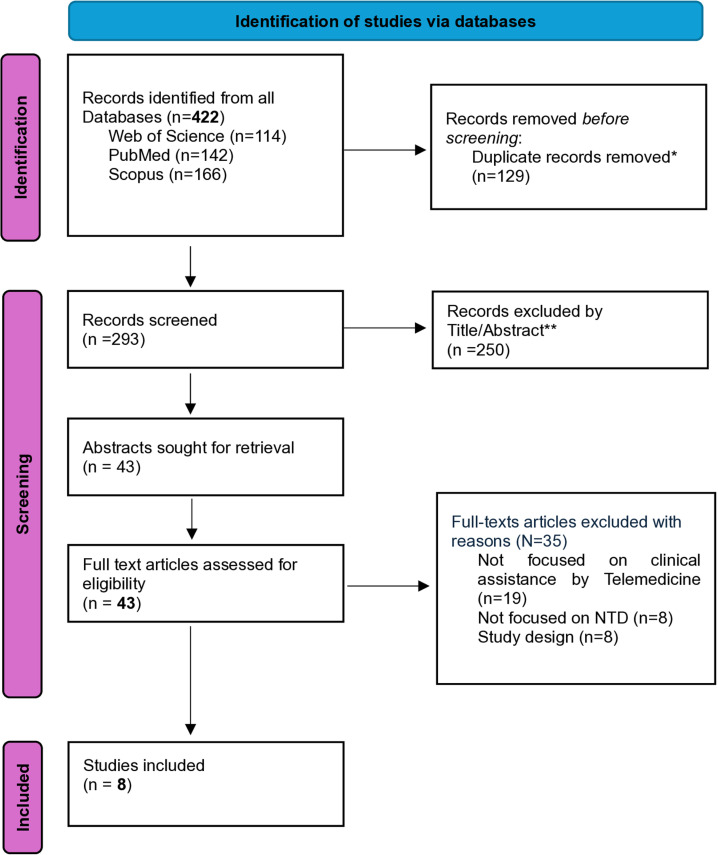
PRISMA–ScR chart. * Duplicates identified and excluded by automation tool *Rayyan* (13); ** abstracts selected by researchers individually in blinded sections and combined using the automation tool *Rayyan* (13).

The survey results and the selection process are full reported in the PRISMA-ScR flow diagram below (Fig 2). A total of 293 relevant articles were eligible for screening after removing duplicates. After screening abstracts and titles, 43 articles were read in full, and 8 articles were selected by eligibility for data extraction, based on the inclusion and exclusion criteria.

#### 1. Summary of studies characteristics.

The analysis of the final selection showed that four studies (N=4) were focused on dermatological diseases [14,15,16,17]: one specifically on leprosy [14] (N=1), one specifically on leishmaniasis [16] (N=1), and two in general dermatological diseases [15,17]. The remaining studies were related to the management of dengue (N=2) [18,19], cysticercosis (N=1) [20] and trachoma (N=1) [21]. The majority of the 21 NTDs were not represented in the results (Table 1)

**Table 1:** Characteristics of the studies included in the Scoping review.

Author/ Reference	Year	Country of healthcare provider	Neglected Tropical Disease (NTD)	Technology/ Telemedicine intervention	Study focus	Major Findings	Total patients and Number of NTD patients who received telemedicine intervention
Trindade et al. [14]	2008	Brazil	Leprosy	Digital Images shared between doctors by Internet-Based System Developed for teledermatology.	Compared the accuracy of the diagnosis sent through images vs. in-person examination in suspected cases of leprosy.	Telemedicine sensitivity:78%; specificity: 31%. Diagnostic agreement: 74%.	N=106 suspect cases of leprosy were evaluated remotely by dermatologists N=59 confirmed for leprosy.
Galván et al. [18]	2014	Paraguay	Dengue	Telephone calls - BONIS system of community telesurveillance of febrile symptoms of dengue. Free software.	Feasibility study; evaluated the results of the implementation of the system.	Increase detection and notification of suspected dengue cases in 157 cases	N=157 suspect dengue cases identified remotely by teleconsultations; N=56 confirmed for dengue.
Konanki et al. [20]	2018	India	Cysticercosis	Telephone calls.	Compared the accuracy of diagnosis by teleconsultation vs. face-to-face to identify critical clinical events in children with neurocysticercosis and seizures after treatment.	Telemedicine sensitivity: 89%; specificity: 97%; 95% of the events did not require face-to-face consultation.	N=228 patients evaluated by phone; N=55 critical clinical complications identified remotely by teleconsultations.
Messagier et al. [15]	2019	French Guyana	Leprosy, Leishmaniasis, Scabies, Tungiasis,	French Guiana Platform. Computer software for telemedicine in primary health care units and dermatologysts in hospitals, with digital camera.	Evaluated the quality and usefulness of the telereferencing system.	92% (n=234) of total cases managed in peripheral health centers, without the need to travel for face-to-face consultations with dermatologists.	N=254 patients with skin diseases evaluated by the telereferencing system; N=29 NTDs cases diagnosed (17 leishmaniasis/7 leprosy/4 escabies/1 tunguiasis)
Naufal et al. [21]	2021	Tanzania. (clinical image classification center in USA)	Trachoma	Image Capture and Processing System (ICAPS). A smartphone photograph and send image to a virtual reading center. Tropicaldata mHealth app record and transmit the datas.	Compared the accuracy of the diagnosis of trachoma eye lesions by teleconsultation vs. face-to-face examination in children.	Agreement between remote grading of the images compared to the grading by presential provider in field was about 95%.	N=1350 patients evaluated for screening; N=54 patients with follicular trachomatous inflammation identified remotely.
Pedrotti et al. [19]	2022	Brazil	Dengue	Videoconferencies - Glob-alMed video-conference software and electronic health records.	Evaluated the effectiveness of teleconsultations in reducing the need for face-to-face medical consultations in patients with mild/moderate acute symptoms during a dengue outbreak.	94.4% of the cases did not require immediate face-to-face consultation.	N=267 confirmed dengue patients evaluated remotely by telemedicine.
Yotsu et al. [17]	2023	Ivory Coast.	Leprosy, Buruli ulcer (BU), Lymphatic filariasis (LF), Scabies, and Yaws	eSkinHealth Mobile app (portable electronic medical record and teledermatology)	Evaluated the app’s effectiveness in rural areas for diagnosing and managing skin NTDs and other skin diseases.	N=151patientswere diagnosed on app.	N=207 skin diseases evaluated on app; N=79 NTDs cases diagnosed (34 scabies/ 26 BU/11 leprosy/4 LF/ 3 yaws/1 mycetoma).
Castillo et al. [16]	2023	Colombia	Leishmaniosis	Mobile app - Guaral ^+^ST app (teledermatology)	Compared the clinical following of leishmaniosis treatment to by telemedicine to face-to-face care.	Telemedicine increased the proportion of patients monitored at week 26 of treatment by around 53%.	N=49 cutaneous leishmaniasis treatments monitored by app.

#### 2. Contexts of telemedicine interventions for NTDs.

Regarding the geographic profile of the populations studied, two researches took place in Brazil and the others were developed in the following countries: Colombia (n=1), French Guyana (n=1), India (n=1), Ivory Coast (n=1), and Paraguai (n=1). Four studies were conducted in countries classified as upper-middle-income economies [22] (Brazil, Colombia, Paraguay), three studies in lower-middle income economies (India, Ivory Coast, Tanzania), and one in a high-income economy country (French Guyana). Four studies were developed at least one of the arms in remote and/or rural areas [2,15–17], three in urban settings [14,18,19] and in one it was not described [20].

The characteristics of the contexts and the telemedicine technologies are presented in Table 2. Five studies had telemedicine providers who were part of the local primary care team (São Paulo, Brazil; Asunción, Paraguay; Tumaco, Colombia; Cayenne, French Guyana; and Sinfra, Ivory Coast), and two of them utilized telemedicine to enable communication between the local team and the hospital [14,15]. One study was carried out only with staff from a tertiary hospital (New Delhi, India); one in a temporary emergency hospital due to a dengue outbreak (São Paulo, Brazil); and one worked in conjunction with a district-level community prevalence research team (Chamwino, Tanzania), which is still the only study where one arm of the clinical assessment was carried out in a different country (United States).

**Table 2:** Thematic framework - characterization of the telemedicine interventions and contexts.

	N	References
**Telemedicine intervention**		
Consultations between remote people and healthcare provider - real-time/synchronous telemedicine	3	[18], [19,20]
Transmission of medical data (e.g. images, notes) mediated by distant healthcare providers - asynchronous telemedicine	5	[14],[15–17,21]
**Communication technology**		
Telephone	2	[18],[20]
Videoconference	1	[19]
Mobile application	3	[16],[17,21]
Data/images submission platform	2	[14],[15]
**Clinical applicability**		
Telemedicine to ensure patient access to diagnostics	6	[14],^15^,^17^,^18^,^20^,^21^]
Telemedicine to ensure patient access to treatments	1	[15],^17^,^19^]
Telemedicine to monitor patient treatment and complications	1	[16],[20],
**Telemedicine providers involved**		
Primary Health Care team	5	[14],[15–18]
Hospital team	4	[14],[15,19,20]
Other	1	[21]
**Location where the study population resides**		
Rural or/and remote	4	[15],[16,17,21]
Urban	3	[14],[18,19]
Not mentioned	1	[20]
SouthAmerica (Brazil, Colombia, French Guyana Paraguai)	5	[14],[15,16,18,19]
Africa (Ivory Coast, Tanzania)	2	[17],[21]
Asia (India)	1	[20]
Upper-middle-income economy	4	[14],[16,18,19]
Lower-middle-income economy	3	[17],[20,21]
High income country	1	[15]
**Disease studied**		
Leprosy	3	[14],[15,17]
Leishmaniosis	2	[15],[16]
Dengue	2	[18],[19]
Scabies	2	[15],[17]
Cysticercosis	1	[20]
Trachoma	1	[21]
Tungiasis	1	[15]
Buruli ulcer, Lymphatic filariasis, Yaws	1	[17]

Only three studies investigated real-time synchronous interventions of consultations between remote patients and healthcare providers, two of which were via ordinary telephone calls [18,20] and only one via videoconference [19]. In all five other articles, telemedicine was carried out with asynchronous transmission of medical data (e.g. images, descriptive clinical notes), involving teleconsultations for case management between healthcare professionals located in different services [14–17,21].

Regarding the telemedicine software used, two studies worked with specific telemedicine platforms for sending clinical data and images [14,15] and one study tested a software for medical videoconferencing [19], while the use of mobile applications for remote care of NTDs occurred in three studies [16,17,21].

#### 3. Main outcomes synthesis of telemedicine in NTDs care.

**Fig3:**
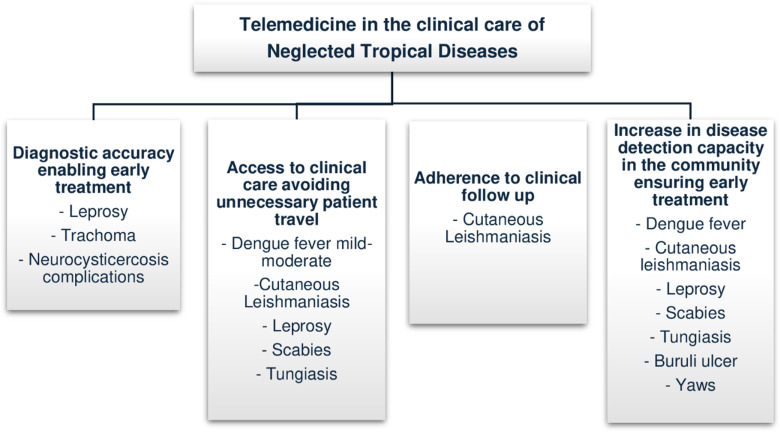
Summary of the main telemedicine benefits for NTD of the scoping review.

A summary of the main evidence of telemedicine benefits obtained in the studies selected for this scoping review is presented in Fig 3. Six studies sought to evaluate the applicability of remote telediagnosis of clinical conditions related to NTD [14,15,17,18,20,21], but only three of them compared the effectiveness/accuracy of remote diagnosis via telemedicine with in-person medical care as a gold standard [14,20,21]. Favorable results have been obtained with the use of teleconsultation in the diagnosis of leprosy (74% of diagnostic agreement) [14], trachoma (95% of diagnostic agreement) [21] and complications of neurocysticercosis (telemedicine sensitivity 89%/specificity 97%) [20], demonstrating high diagnostic accuracy in the use of remote resources for these conditions.

One study found a significant increase in dengue fever detection capacity of the primary care service after implementing remote surveillance of suspected symptoms in the community, with a documented increase in the reporting of suspected cases compared to the previous two years (157 more cases) [18]. An increase on diagnostic and treatment capacity of skin NTDs in communities was also observed in two studies [15,17] in which specialist physicians assessed and followed via telemedicine cases of leprosy, Buruli ulcer, yaws, leishmaniasis, scabies, and tungiasis.

Another primary result on the use of telemedicine found in the studies was a significant reduction in the need for medical in-person specialized consultations for the care of mild and moderate dengue fever suspected cases (94,4%/n=252) [19] and some dermatological NTDs [15], avoiding the need for patients to travel to distant health services and enabling the introduction of early treatment.

Clinical monitoring by telephone for tracking crises in patients with treated neurocysticercosis has proven to be a relevant strategy for remote monitoring of complications of this chronic disease, allowing the necessary clinical follow-up without the need for sophisticated technology [20]. Telemedicine also proved useful in improving clinical monitoring of cutaneous leishmaniasis in one of the studies, in which patients monitored remotely via a mobile application had better adherence to clinical follow-up than patients who were only monitored in person at the service [16].

## Discussion

This scoping review allowed the general mapping of available scientific production related to NTD care via telemedicine. The majority of the 21 NTDs were not represented in the results. The study demonstrated that there is a gap in recommendations for the clinical management of acute and chronic NTDs through telemedicine, revealing that this resource is still largely underutilized for the care of this group of infectious diseases of poverty. By way of comparison with a single other infectious disease, a bibliometric study published in May 2022 that investigated the application of telemedicine in the care of COVID-19 retrieved 5.224 research papers published up to that time [23]. A number 12,37 times greater than what we found for the 21 NTDs together until 2024. None of the selected studies were conducted in a country with a low-income economy, suggesting that the challenges of implementing remote care services in extreme poverty contexts are a relevant gap [4]. The integration of digital health systems into care networks is a challenge in health systems with few resources, as their effectiveness depends on local infrastructure, internet accessibility and connection quality, in addition to disruptive changes in the existing healthcare routine [3,4].

### Remote care for neglected tropical diseases

Cutaneous NTDs stood out for their applicability in remote clinical approaches. The main findings in this review around teledermatology corroborate the evidence on the previous consolidation of this specialty in the international telehealth scenario, including NTDs [24,25]. The ease of managing clinical parameters in dermatology through photographs and videos, increasingly accessible on modern devices seems to facilitate. The use of mobile phones for healthcare (mobile health, currently called mHealth) was also shown to be more focused on neglected skin diseases than other NTDs in our study results. It is important to highlight, however, that there are also limitations that make substitutive and exclusive longitudinal follow-up via telemedicine impossible for some diseases. For leprosy and cutaneous leishmaniasis, for example, the mucosal lesions would be difficult to verify through photographs and would require in-person visits to the service.

Dengue is an NTD transmitted by mosquitoes that can quickly evolve into severity and overwhelm emergency services in local outbreaks. The use of telemedicine in dengue outbreaks has emerged as an effective strategy for early diagnosis and medical assessment of low-to-moderate risk cases, expanding case identification and preventing progression to severity. However, the urgency of the global epidemiological scenario of the disease [26] suggests that this resource could be more often used by health systems in order to avoid preventable deaths in areas with limited health service coverage. Digital health consultations combined with point-of-care diagnostic methods, such as rapid tests, is a window of opportunity to expand the differential diagnosis of acute febrile illnesses in the large endemic regions of South America and Southeast Asia and may also be useful in African regions where there is the challenge of differential diagnosis with malaria.

Trachoma is an infectious eye disease and an important cause of blindness in poor, rural and remote areas of the world. In our review we observed that the integration between screening programs and remote diagnosis by telemedicine can be a feasible advance in its elimination plan. One study uses a mHealth application suggesting an advance incorporation of mobile health resources in trachoma care [21]. However, no telehealth actions focused on preventive chemotherapy for trachoma in endemic communities were identified in our review. The development of integrated preventive chemotherapy telemonitoring actions could be beneficial to support community strategies for the control and elimination of other NTDs, such as onchocerciasis, lymphatic filariasis, schistosomiasis and soil-transmitted helminthiasis in isolated communities at risk.

For some long-term NTDs typical of remote rural areas, such as Chagas Disease and Human African Trypanosomiasis, no results were recovered, although it was expected that continuous chronic telemonitoring of these patients could have good applicability, as it would avoid unnecessary trips to distant specialized centers for clinical monitoring of complications. Despite the advancement of the international debate on One Health approaches, the clinical management of some zoonotic NTDs seem to out of sight by the global digital health agenda, with technological advances more concentrated in sectors like vectors surveillance [27,28] or digital microbiology [29].

### Telemedicine scenarios

Most interventions took place in South America. We identified a shortage of studies in African countries and the lack of innovative health experiences aimed at this region is a permanent challenge in controlling NTDs. Although the African continent accounts for around 40% of the global NTD burden [30], its broadband internet penetration rate was still around 33% in 2020 [31]. Applications that work offline and do not require constant internet can be part of the solution, but the challenge of providing access to person-centered services of remote healthcare especially in the sub-Saharan region requires complex multisectoral efforts and local engagement. The shortage of skilled health workforce in this region is a critical problem for addressing prevalent diseases, and consultations with specialists located in distant centers may be a good alternative in supporting infectious disease care [32]. Furthermore, investment in public health systems with broad coverage can contribute to decentralized care in communities affected by NTDs but also requires local governance policies committed to this agenda.

NTDs are closely related to the territory’s environmental health conditions, and telemedicine applied at the level of primary health care, as seen in five of the studies [14,15,16,17,18] has the potential to expand digital community strategies for local surveillance and better epidemiological control. Improving the management of clinical communication between different levels of care (e.g. primary care centers and tertiary hospitals) through integrated telemedicine platforms can also bring potential benefits to continuity, comprehensiveness and clinical coordination, considered internationally as important pillars for strengthening health systems centered on primary care [33]. These results are also important to strengthen joint action between general practitioners and focal specialists through telemedicine in the management of patients with NTDs.

### The implementation of telemedicine

The length of the telemedicine studies selected ranged from 2 to 18 months. Lack of results on long-term telemedicine implementations limits the understanding of the real barriers to its incorporation into services. As most of the studies identified in this review consisted of proofs of concept or pilot tests, it would be important to investigate which initiatives remained in operation, to assess the factors that may be associated with their success and sustainability in real-world settings over time.

On the way to mitigate global inequities in access to internet and communication/information technologies in health, the implementation of telemedicine on a large scale requires complex efforts from several areas for its incorporation into health services: legal rules; systems interoperability; person-centered clinical telemedicine protocols; specific training for health providers; and promotion of patients’ digital literacy - which can be difficult for populations with low socioeconomic status. Another aspect that should be considered in telemedicine aimed at NTDs is digital security and ethical regulation practices [3,4,31–33]. Concern about ethical care when sending sensitive health data virtually is mandatory in the development of safe telemedicine tools in poor countries, that sometimes have limited legislation to guarantee the data protection of vulnerable populations.

The temporal analysis demonstrated that 50% of the studies (n=4) were published between 2021 and 2023, following the recent international trend of growing interest in digital health technologies [34]. But most of the initiatives presented in the studies occurred asynchronously or via simple phone calls. Thus, the benefits of implementing synchronous teleconsultations with video calls for the diagnosis and treatment of NTDs was therefore a knowledge gap identified by this review. Only one study presented a structured telemedicine platform that offered teleconsultation via videoconference in real time between patients and doctors, but it did not eliminate the need for the patient to travel to the health service, since the medical teleconsultation took place after an in-person assessment by nursing staff [19]. Henceforth, the use of mobile applications for remote care for NTDs emerges as a promising resource [16,17,21].

### Limitations

No formal quality assessment of the studies included was performed, as scoping reviews present a map of what evidence has been produced, rather than only searching for the best available evidence to answer a specific question.

Publication bias is another limitation of this work, as we only considered articles published in journals and the search did not cover grey literature. To overcome this problem, we used broader search strategies to map local experiences originating especially in low-income settings. As NTD constitutes a clinically diverse group of diseases, with different durations, stages of evolution and possible complications, in-depth comparative variables for intervention approaches are scarce. However, we highlight the main common characteristics and interventions aspects.

The group of neglected fungal diseases is not specified in the WHO list, described as “mycetoma, chromoblastomycosis and other deep mycoses” [12], making it difficult to include each of these diseases in the search strategy (Fig 1), which may contribute to selection bias. For example, although paracoccidiomycosis and histoplasmosis are relevant problems in the Americas, we did not find consistent evidence to support the nominal inclusion of these pathologies in our review search strategy [35,36].

## Conclusion

Telemedicine applied to NTDs is not a new field, but the results demonstrate that the use is limited compared to the global burden of NTDs. Although there is a recent increase in academic production on the topic, the evidence is restricted to a small group of diseases and a few countries, and the technologies used were, in most cases, asynchronous. The impacts of using this modality of care likely depend on the disease being addressed, the local context and the stage of clinical follow-up. Our results illustrate the importance of further research into clinical benefits of virtual care applied to each NTD.

Remote resources have emerged globally as a promise for reducing disparities in access to healthcare services, especially in geographically isolated areas. Telemedicine has the potential to increase access to early care, optimize the efficiency of follow-up visits and travel times, and improve communication between primary care providers and distant hospitals. Although its popularity has grown, the research gap regarding its effectiveness in the clinical treatment of each NTDs is still large.

The limited data available in this review suggests that telemedicine is feasible, but further in-depth studies are needed to determine the level of quality of current evidence and the benefits of this modality. Cost-effectiveness results are also needed to support decision-makers, especially in poor healthcare settings.

## References

[1] World Health Organization. Ending the neglect to attain the Sustainable Development Goals: a road map for neglected tropical diseases 2021–2030. Geneva: World Health Organization; 2021. Available from: https://www.who.int/teams/control-of-neglected-tropical-diseases/ending-ntds-together-towards-2030.

[2] World Health Organization. Global report on neglected tropical diseases 2023. Geneva: World Health Organization; 2023. Available from: https://www.who.int/teams/control-of-neglected-tropical-diseases/global-report-on-neglected-tropical-diseases-2023

[3] World Health Organization. Global diffusion of eHealth: Making universal health coverage achievable. Report of the third global survey on eHealth. Geneva: WHO Global Observatory for eHealth; 2016. Available from: https://apps.who.int/iris/bitstream/handle/10665/252529/9789241511780-eng.pdf?sequence=1.

[4] World Health Organization. Global strategy on digital health 2020-2025. Geneva: World Health Organization; 2020. Available from: https://www.who.int/publications/i/item/9789240020924

[5] Tilahun B, Gashu KD, Mekonnen ZA, Endehabtu BF, Angaw DA. Mapping the role of digital health technologies in the case detection, management, and treatment outcomes of neglected tropical diseases: a scoping review. Trop Med Health. 2021;49(1):17. doi: 10.1186/s41182-021-00307-1 33618757 PMC7898439

[6] Dorsey ER, Topol EJ. Telemedicine 2020 and the next decade. Lancet. 2020;395(10227):859. doi: 10.1016/S0140-6736(20)30424-4 32171399

[7] Mahmoud K, Jaramillo C, Barteit S. Telemedicine in low- and middle-income countries during the COVID-19 pandemic: a scoping review. Front Public Health. 2022;10:914423. doi: 10.3389/fpubh.2022.914423 35812479 PMC9257012

[8] World Health Organization. Classification of digital interventions, services and applications in health: a shared language to describe the uses of digital technology for health, 2nd ed. Geneva: World Health Organization; 2021. Available from: https://www.who.int/publications/i/item/9789240081949

[9] Ghassemi M, Oakden-Rayner L, Beam AL. The false hope of current approaches to explainable artificial intelligence in health care. Lancet Digit Health. 2021;3(11):e745–50. doi: 10.1016/S2589-7500(21)00208-9 34711379

[10] Aromataris E. Munn Z. Joanna Briggs Institute Reviewer’s Manual. Adelaide: The Joanna Briggs Institute; 2020.

[11] Tricco AC, Lillie E, Zarin W, O’Brien KK, Colquhoun H, Levac D, et al. PRISMA extension for scoping reviews (PRISMA-ScR): checklist and explanation. Ann Intern Med. 2018;169(7):467–73. doi: 10.7326/M18-0850 30178033

[12] World Health Organization. Neglected tropical diseases. [Updated 2023 Feb 22]. Available from: https://www.who.int/health-topics/neglected-tropical-diseases#tab=tab_1

[13] Ouzzani M, Hammady H, Fedorowicz Z, Elmagarmid A. Rayyan-a web and mobile app for systematic reviews. Syst Rev. 2016;5(1):210. doi: 10.1186/s13643-016-0384-4 27919275 PMC5139140

[14] Trindade MAB, Wen CL, Neto CF, Escuder MM, Andrade VLG, Yamashitafuji TMT, et al. Accuracy of store-and-forward diagnosis in leprosy. J Telemed Telecare. 2008;14(4):208–10. doi: 10.1258/jtt.2008.071203 18534956

[15] Messagier A-L, Blaizot R, Couppié P, Delaigue S. Teledermatology use in remote areas of french guiana: experience from a long-running system. Front Public Health. 2019;7:387. doi: 10.3389/fpubh.2019.00387 31921751 PMC6930889

[16] Castillo M, Alexander N, Rubiano L, Rojas C, Navarro A, Rincon D, et al. Randomized trial evaluating an mHealth intervention for the early community-based detection and follow-up of cutaneous leishmaniasis in rural Colombia. PLoS Negl Trop Dis. 2023;17(3):e0011180. doi: 10.1371/journal.pntd.0011180 36972285 PMC10079216

[17] Yotsu RR, Almamy D, Vagamon B, Ugai K, Itoh S, Koffi YD, et al. An mHealth App (eSkinHealth) for detecting and managing skin diseases in resource-limited settings: mixed methods Pilot Study. JMIR Dermatol. 2023;6:e46295. doi: 10.2196/46295 37632977 PMC10335127

[18] Galván P, Cane V, Samudio M, Cabello A, Cabral M, Basogain X, et al. Implementation of a community tele-epidemiological surveillance system using information and communication technologies in Paraguay. Rev Panam Salud Publica. 2014;35(5–6):353–8. 25211561

[19] Pedrotti CHS, Accorsi TAD, Moreira FT, Lima KDA, Köhler KF, Gaz MVB, et al. Telemedicine medical evaluation of low-risk patients with dengue during an outbreak may be an option in reducing the need for on-site physicians. Int J Infect Dis. 2022;121:106–11. doi: 10.1016/j.ijid.2022.04.059 35504552

[20] Gulati S, Shruthi NM, Panda PK, Sharawat IK, Josey M, Pandey RM. Telephone-based follow-up of children with epilepsy: Comparison of accuracy between a specialty nurse and a pediatric neurology fellow. Seizure. 2020;83:98–103. doi: 10.1016/j.seizure.2020.10.002 33120328 PMC7536121

[21] Naufal F, Brady CJ, Wolle MA, Saheb Kashaf M, Mkocha H, Bradley C, et al. Evaluation of photography using head-mounted display technology (ICAPS) for district Trachoma surveys. PLoS Negl Trop Dis. 2021;15(11):e0009928. doi: 10.1371/journal.pntd.0009928 34748543 PMC8601615

[22] World Bank. (2024). World Bank country and lending groups [Data file]. Retrieved from https://datahelpdesk.worldbank.org/knowledgebase/articles/906519-world-bank-country-and-lending-groups [Acessed in Jun 01, 2024]

[23] Lan X, Yu H, Cui L. Application of telemedicine in COVID-19: a bibliometric analysis. Front Public Health. 2022;10:908756. doi: 10.3389/fpubh.2022.908756 35719666 PMC9199898

[24] Carrion C, Robles N, Sola-Morales O, Aymerich M, Ruiz Postigo JA. Mobile health strategies to tackle skin neglected tropical diseases with recommendations from innovative experiences: systematic review. JMIR Mhealth Uhealth. 2020;8(12):e22478. doi: 10.2196/22478 33382382 PMC7808891

[25] Yotsu R, Ding Z, Hamm J, Blanton R. Deep learning for AI-based diagnosis of skin-related neglected tropical diseases: a pilot study. medRxiv. 2023:2023.03.14.23287243. doi: 10.1101/2023.03.14.23287243 37578966 PMC10449179

[26] Pan American Health Organization. Situation Report No 20 - Dengue Epidemiological Situation in the Region of the Americas - Epidemiological Week 20, 2024. Washington, D.C.: PAHO; 2024. Available in: https://www.paho.org/en/documents/situation-report-no-20-dengue-epidemiological-situation-region-americas-epidemiological

[27] Delgado-Noguera LA, Hernández-Pereira CE, Ramírez JD, Hernández C, Velasquez-Ortíz N, Clavijo J, et al. Tele-entomology and tele-parasitology: A citizen science-based approach for surveillance and control of Chagas disease in Venezuela. Parasite Epidemiol Control. 2022;19:e00273. doi: 10.1016/j.parepi.2022.e00273 36118050 PMC9475302

[28] Lwin MO, Jayasundar K, Sheldenkar A, Wijayamuni R, Wimalaratne P, Ernst KC, et al. Lessons from the implementation of mo-buzz, a mobile pandemic surveillance system for dengue. JMIR Public Health Surveill. 2017;3(4):e65. doi: 10.2196/publichealth.7376 28970191 PMC5643840

[29] Hasker E, Kwete J, Inocencio da Luz R, Mpanya A, Bebronne N, Makabuza J, et al. Innovative digital technologies for quality assurance of diagnosis of human African trypanosomiasis. PLoS Negl Trop Dis. 2018;12(9):e0006664. doi: 10.1371/journal.pntd.0006664 30212459 PMC6136689

[30] World Health Organization Regional Office for Africa. Promising Progress on Neglected Tropical Diseases in Africa [Internet]. World Health Organization; 2022. Available from: https://www.afro.who.int/news/promising-progress-neglected-tropical-diseases-africa

[31] World Health Organization. Regional Committee for Africa, 71. Framework for Implementing the Global Strategy on Digital Health in the African Region. World Health Organization Regional Office for Africa; 2021. Available from: https://iris.who.int/handle/10665/348981.

[32] Dodoo JE, Al-Samarraie H, Alsswey A. The development of telemedicine programs in Sub-Saharan Africa: Progress and associated challenges. Health Technol (Berl). 2022;12(1):33–46. doi: 10.1007/s12553-021-00626-7 34849325 PMC8613515

[33] Hone T, Macinko J, Millett C. Revisiting Alma-Ata: what is the role of primary health care in achieving the Sustainable Development Goals?. Lancet. 2018;392(10156):1461–72. doi: 10.1016/S0140-6736(18)31829-4 30343860

[34] Maugeri A, Barchitta M, Basile G, Agodi A. Public and Research interest in telemedicine from 2017 to 2022: infodemiology study of google trends data and bibliometric analysis of scientific literature. J Med Internet Res. 2024;26:e50088. doi: 10.2196/50088 38753427 PMC11140276

[35] Griffiths J, Lopes Colombo A, Denning DW. The case for paracoccidioidomycosis to be accepted as a neglected tropical (fungal) disease. PLoS Negl Trop Dis. 2019;13(5):e0007195. doi: 10.1371/journal.pntd.0007195 31095569 PMC6522030

[36] Oladele RO, Ayanlowo OO, Richardson MD, Denning DW. Histoplasmosis in Africa: An emerging or a neglected disease?. PLoS Negl Trop Dis. 2018;12(1):e0006046. doi: 10.1371/journal.pntd.0006046 29346384 PMC5773084

